# The diversity effect in inductive reasoning depends on sampling assumptions

**DOI:** 10.3758/s13423-018-1562-2

**Published:** 2019-01-25

**Authors:** Brett K. Hayes, Danielle J. Navarro, Rachel G. Stephens, Keith Ransom, Natali Dilevski

**Affiliations:** 1 School of Psychology, University of New South Wales, Sydney, NSW 2052 Australia; 2 School of Psychology, University of Adelaide, Adelaide, SA 5005 Australia

**Keywords:** Category-based induction, Evidence diversity, Bayesian modeling, Relevance theory, Sampling assumptions

## Abstract

A key phenomenon in inductive reasoning is the diversity effect, whereby a novel property is more likely to be generalized when it is shared by an evidence sample composed of diverse instances than a sample composed of similar instances. We outline a Bayesian model and an experimental study that show that the diversity effect depends on the assumption that samples of evidence were selected by a helpful agent (strong sampling). Inductive arguments with premises containing either diverse or nondiverse evidence samples were presented under different sampling conditions, where instructions and filler items indicated that the samples were selected intentionally (strong sampling) or randomly (weak sampling). A robust diversity effect was found under strong sampling, but was attenuated under weak sampling. As predicted by our Bayesian model, the largest effect of sampling was on arguments with nondiverse evidence, where strong sampling led to more restricted generalization than weak sampling. These results show that the characteristics of evidence that are deemed relevant to an inductive reasoning problem depend on beliefs about how the evidence was generated.

**Electronic supplementary material:**

The online version of this article (10.3758/s13423-018-1562-2) contains supplementary material, which is available to authorized users.

Philosophers of science have suggested that diverse evidence leads to more robust generalization (e.g., Hempel, 1966). The “diversity effect” in category-based induction suggests that most adults share this intuition: people are more likely to generalize a novel property to other category members when that property is shared by a diverse set of categories rather than a nondiverse set. For example, knowing that lions and cows have some property *p* is generally seen as a stronger basis for generalizing that property to other mammals than knowing that lions and tigers have property *p* (Osherson, Smith, Wilkie, Lopez, & Shafir, 1990). This diversity effect is robust, having been replicated across a range of reasoning tasks and category stimuli (e.g., Feeney & Heit, 2011; Liew, Grisham, & Hayes, 2018; Osherson et al., 1990). Moreover, diverse samples of evidence have been shown to facilitate hypothesis testing (e.g., López, 1995) and promote conceptual change (Hayes, Goodhew, Heit, & Gillan, 2003). Early accounts of the diversity effect in category-based induction emphasized the crucial role of similarity between those categories known to have a property (premise categories) and the categories to which the property could be generalized (conclusion categories). Osherson et al.’s (1990) influential similarity-coverage model, for example, attributes the diversity effect to the fact that diverse premise categories (e.g., lions and cows) have greater “coverage” of broader conclusion categories such as mammals (i.e., diverse premise categories are similar to more members of a superordinate like mammals than are nondiverse categories).

There is a growing consensus in the field, however, that similarity alone is insufficient to explain property induction (e.g., Kemp & Tenenbaum, 2009; Medin, Coley, Storms, & Hayes, 2003). Inductive arguments involving premise and conclusion categories (e.g., lions and cows have *p*, therefore mammals have *p*) are often communicative acts, designed to influence the beliefs of the reasoner, and as such, pragmatic inferences can shape the perceived strength of the inductive argument (Goodman & Frank, 2016; Grice, 1975). Experimental manipulations of the communicative context influence how people interpret an inductive argument (Ransom, Perfors, & Navarro, 2016; Voorspoels, Navarro, Perfors, Ransom, & Storms, 2015), in a manner consistent with Bayesian theories of inductive reasoning (Navarro, Dry, & Lee, 2012; Sanjana & Tenenbaum, 2003; Tenenbaum & Griffiths, 2001). Within the Bayesian framework, these effects are seen as reflecting changes in sampling assumptions—assumptions that a reasoner makes about how an inductive argument was constructed.

Much of the literature on sampling assumptions has focused on the effect of adding new evidence (e.g., additional premise categories) to an inductive argument (e.g., Fernbach, 2006; Ransom et al., 2016). However, to the extent that these findings reflect the operation of more general principles of Bayesian reasoning (Sanjana & Tenenbaum, 2003; Tenenbaum & Griffiths, 2001), one might wonder if sampling assumptions also shape the value people assign to the diversity of evidence in inductive arguments. Our goal in this article is to address this question. Is the diversity effect in inductive reasoning purely a similarity-driven effect, or does it depend on how the reasoner believes the inductive argument was constructed?

## Reasoning as Bayesian inference

The Bayesian perspective on inductive reasoning asserts that human reasoning can be viewed as a form of probabilistic inference (Kemp & Tenenbaum, 2009; Sanjana & Tenenbaum, 2003). Consider an inductive argument whose premises assert that the categories *x* = (*x*_1_, . . . , *x*_*n*_) possess property *p*. When asked to assess the evidence for some hypothesis *h* about which categories share the property in light of the evidence ***x*** presented in an argument, the learner reasons as follows. Based on their preexisting knowledge of the world, the reasoner initially assigns some *prior* degree of plausibility *P*(*h*) to the claim. This prior belief *P*(*h*) is updated via Bayes rule to a *posterior* belief *P*(*h*|***x***) that takes account of the evidence, as follows:

$$ P\left(h|x\right)=\frac{P\left(x|h\right)\kern0.3em P(h)}{\sum_{h^{\prime }}P\left(x|{h}^{\prime}\right)\kern0.3em P\left({h}^{\prime}\right)} $$

The central characteristic of this belief revision is that it is driven by the likelihood *P*(*x*|*h*) that the reasoner would have encountered the evidence *x* if the hypothesis *h* correctly described the true extension of the property *p*. Importantly, this likelihood is subjective: It is based on the reasoner’s personal theory about how the inductive argument was constructed, referred to as the sampling assumption (e.g., Fernbach, 2006; Navarro et al., 2012; Tenenbaum & Griffiths, 2001).

**Fig. 1:**
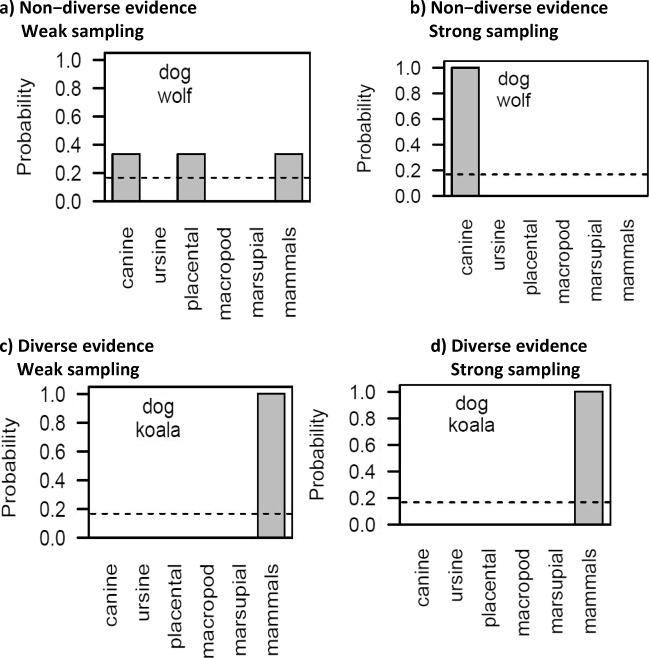
Bayesian reasoning on the example problem. We assume a uniform prior over six hypotheses (dashed line) about which mammal categories have a property *p* (*P*(*h*) = 1/6), and approximately accurate knowledge of the real-world size of each category: canines (|*h*| = 36), ursines (|*h*| = 8), all placentals (|*h*| = 4,000), macropods (|*h*| = 59), all marsupials (|*h*| = 334) and all mammals (|*h*| = 5,000). This toy model highlights the key qualitative constraint: When the evidence is nondiverse, the willingness to generalize to a superordinate depends on sampling assumptions. Under strong sampling, nondiverse evidence will lead to a marked reduction in generalization to the superordinate (panel **b**). Under weak sampling, this reduction will be smaller (panel **a**). However, when evidence is diverse (panels **c** and **d**), the willingness to endorse a superordinate category (mammals) should be high regardless of how the evidence was selected (strong or weak sampling)

To illustrate the workings of the Bayesian model, consider a simple reasoning problem. Suppose a reasoner is told about a novel biological property *p* (e.g., leptine) and asked to infer which species of animals possess the property. Plausible hypotheses *h* might correspond to categories at varying levels in a taxonomic hierarchy. For simplicity, we suppose that the learner considers the six mammal categories listed in Fig. 1, and that all six are deemed equally plausible a priori (hence, *P*(*h*) = 1/6). We further assume that combinations of categories (e.g., canines and ursines) are not entertained.

A key implication of our approach is that sampling assumptions matter more for inferences based on nondiverse evidence. To illustrate, suppose that the learner is now told that dogs and wolves both produce leptine. How should a Bayesian reasoner behave? The answer depends on what the reasoner believes about why they were informed about dogs and wolves, specifically. One possibility—known as weak sampling—is that these two animals were chosen at random, and by chance it happened to be two canines, and (also by chance) the two canines do produce leptine. Because the items are chosen at random, irrespective of whether or not they have the property in question, the likelihood takes on a constant value *P*(*x*|*h*) ∝ 1 for every hypothesis that is consistent with the evidence (i.e., canines, placentals, mammals), and *P*(*x*|*h*) = 0 for all hypotheses that are not (ursines, macropods, marsupials). The posterior distribution is therefore evenly spread across the three still-plausible hypotheses—that is, *P*(*h*|*x*) = 1/3 (see Fig. 1a).

Another alternative in the literature is known as strong sampling, and describes situations where the premise categories *x* are selected precisely because they possess the property *p*. Perhaps a helpful teacher looked up a list of leptine-producing animals and then randomly chose two illustrative animal items from this list (e.g., dog and wolf). This produces a model in which the probability of sampling item *x* is given by *P*(*x*|*h*) = 1/|*h*|, where |*h*| denotes the size of the hypothesis. Importantly, this leads to a change in the reasoning process. If the learner believes there are 36 species of canine in the list, then for *h* = canines, the probability of choosing a wolf is 1/36, and the probability of choosing a wolf and a dog (without replacement) is 1/36 × 1/35 ≈ 7.9 × 10^−4^. In contrast, if the true extension of the category is all mammals (*h* = mammals), the chance of selecting a wolf and a dog is extremely small, say 1/5,000 × 1/4,999 ≈ 4.0 − 10^−8^. Taking the ratio of these two probabilities, *P*(wolf, dog | canines) : *P*(wolf, dog | mammals) = 7.9 × 10^−4^ : 4.0 × 10^−8^ ≈ 19,837:1, we see that the evidence is much more likely under the smaller hypothesis (*h* = canines). Repeating the exercise for the case of canines versus placentals, we find a similarly large ratio. That is, *P*(wolf, dog | canines) : *P*(wolf, dog | placentals) = 7.9 − 10^-4^ : 6.3 × 10^-8^ ≈ 12,692:1 Thus, after eliminating those hypotheses that are inconsistent with the evidence (ursines, macropods, and marsupials), the posterior distribution overwhelmingly favors the canine hypothesis over the placental or mammal hypothesis (see Fig. 1b). Specifically, *P*(canines | wolf, dog) = (0.16 × 7.9 * 10^−4^) / ((0.16 × 7.9 × 10^−4^) + (0.16 × 4.0 × 10^−8^) + (0.16 × 6.3 × 10^−8^)) ≈ 0.99. By comparison, *P*(mammals | wolf, dog) ≈ 5.0 ×10^−5^, and *P*(placentals | wolf, dog) ≈ 7.9 ×10^−5^. The strong sampling model therefore embodies a size principle in which the reasoner comes to prefer the smallest or most specific hypothesis that is consistent with the evidence.

To illustrate the implications for the diversity effect, consider how the previous example plays out if the reasoner is given diverse evidence, say, that dogs and koalas produce leptine. In this situation, the sampling model is largely irrelevant: The evidence is only consistent with a single hypothesis (mammals), so the reasoner will strongly endorse an argument generalizing from dogs and koalas to all mammals, regardless of the sampling assumption (see Fig. 1c–d). This leads to our key prediction about the impact of sampling assumptions on the diversity effect—the effect will be far larger under strong sampling assumptions (compare Fig. 1b and d) than under weak sampling assumptions (compare Fig. 1a and c).

**Fig. 2:**
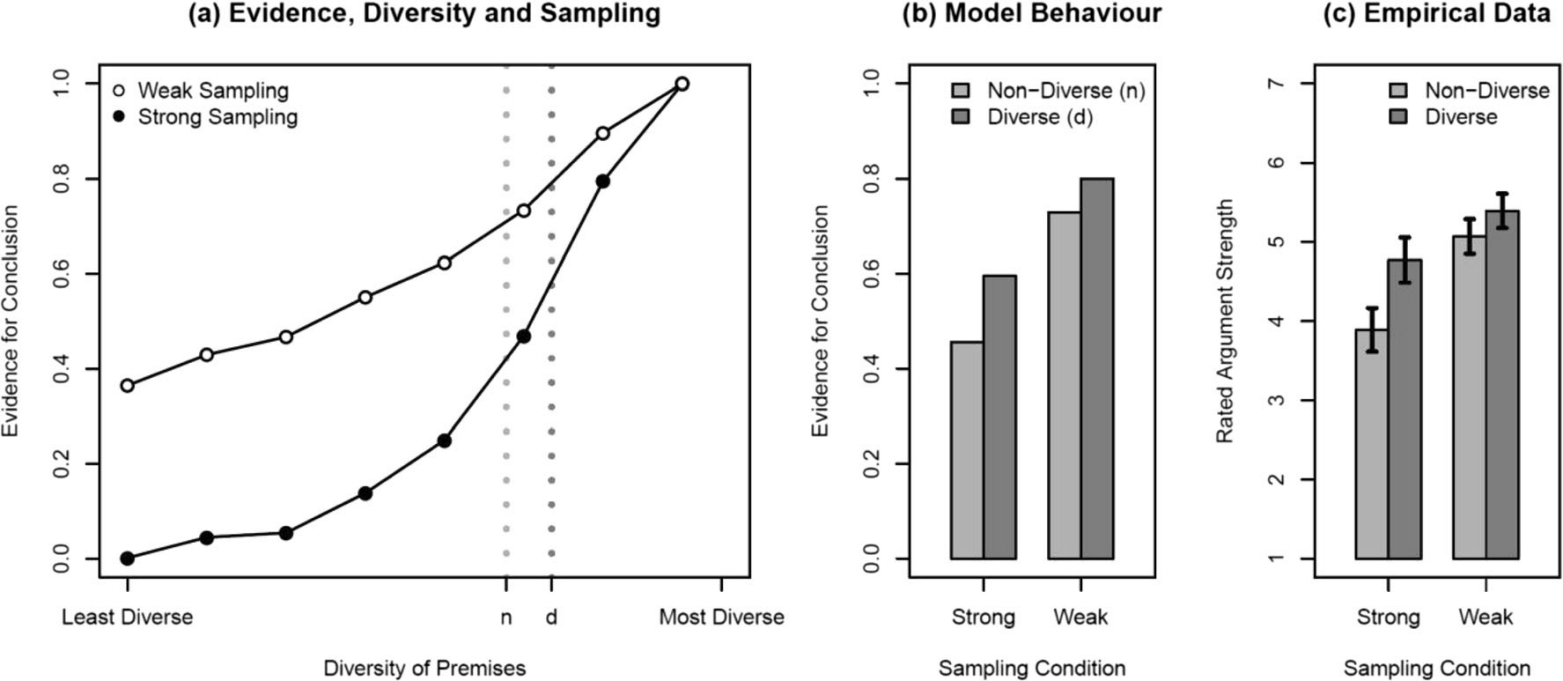
Predicted interaction between premise diversity and sampling type based on our simulation (panel **a**), qualitative predictions derived from the simulation (panel **b**), and the empirical data (panel **c**). Panel **c** plots the mean ratings, and error bars depict standard errors. To produce the model prediction in Fig. 2b from the curves in Fig. 2a, we assumed that there was some latent “perceived” diversity for the premises in the diverse conditions (d) and the nondiverse conditions (n) in our experiment. We estimated these parameters by minimizing sum squared error between empirical means and model generalizations (see Supplementary Materials for details)

Moreover, a simulation of diversity effects under strong or weak sampling over a larger and more general hypothesis space showed that this is a generic prediction of the Bayesian framework (see Supplementary Materials for simulation details and https://osf.io/fpx9k/ for the simulation code). The simulation results shown in Fig. 2a show that both weak and strong sampling models predict a diversity effect (i.e., higher evidence for property generalization to a superordinate conclusion category with more diverse as compared with less diverse premises), but the effect is more pronounced under strong sampling, as indicated by the steeper curve. A notable but perhaps less obvious prediction from this model is that, overall, we should see stronger generalization to a superordinate under weak sampling than under strong sampling.

## Experiment

We carried out an experimental test of these predictions in a property-induction experiment in which target arguments containing diverse or nondiverse premises were presented to groups under conditions that promoted an assumption of either strong or weak sampling. Each group received instructions that described the process by which premises were selected (selected by a helpful agent vs. selected randomly), together with a set of filler arguments, designed to reinforce this description. In the strong sampling group, fillers resembled target items and contained diverse and nondiverse arguments with the same conclusion category. In the weak sampling group, the fillers conveyed the impression that the premises had been generated randomly. This combination of instructional and item manipulation has been successful in previous work in shifting people toward a belief in strong or weak sampling (Ransom et al., 2016; Voorspoels et al., 2015), and has been more effective than cover-story manipulations alone (see Navarro et al., 2012).

### Participants

One hundred and eighty-seven participants from the United States were recruited through Amazon Mechanical Turk (AMT) and paid US$1.00. All had high approval status (≥95% approval for previous tasks). Three were excluded because they failed the attention check administered at the end of the procedures (see below for details). The final sample total was 184 (81 female, 103 male; age: *M* = 35.97 years, *SD* = 10.92), with equal numbers randomly assigned to strong or weak sampling groups.

### Materials

In each sampling condition, 12 arguments were constructed as shown in Table 1. Each argument contained three premise categories and a more general conclusion category, all drawn from the domain of living things. The same six target arguments were presented to each sampling group, half with diverse premises and half with nondiverse premises (see Table 1a–b). Diverse and nondiverse versions of each argument had the same conclusion.

**Table 1:** The inductive arguments used in the task

(a) Target arguments (diverse)		(b) Target arguments (nondiverse)	
dogs, rats, whales → all mammals		rabbits, raccoons, squirrels → all mammals	
octopi, eels, trout → all sea creatures		sardines, herring, anchovies → all sea creatures	
flies, termites, millipedes → all insects		bees, wasps, hornets → all insects	
(c) Filler arguments (strong sampling condition)			
cows, mice, seals → all mammals		zebras, giraffes, camels → all mammals	
pigeons, hens, ostriches → all birds		ducks, swans, pelicans → all birds	
apples, peaches, papaya → all fruit		strawberries, blueberries, raspberries → all fruit	
(d) Filler arguments (weak sampling condition)			
chickens, condors, coconuts → all mammals		geese, skunks, ¬ carp → all mammals	
elephants, moths, pineapples → all birds		robins, salmon, ¬ cod → all sea creatures	
spiders, finches, ¬ worms → all insects		¬ tigers, ¬ bananas, locusts → all fruit	
(e) List of properties used			
leptine	biotin	protein K12	pyroxene
sarca	the chemical didymium	dihedron	enzyme J6
traces of magnesium	actone	bynein	lutein

Because property induction is affected by the typicality of premises (i.e., the extent to which each premise category is seen as representative of the broader conclusion category; Osherson et al., 1990), it was important that this was controlled. Premises for target arguments were chosen in order to match the mean premise typicality across diverse and nondiverse versions, as rated by 162 participants recruited through AMT who were paid US$0.50 but did not participate in the main study.

The two sampling groups received six different filler items. In strong sampling, the fillers were three arguments with diverse premises and three with nondiverse premises (see Table 1c for examples). In weak sampling, each filler contained three premises, drawn from two or three different superordinate categories of living things (see Table 1d for examples). To further reinforce the impression of randomness, four of the six fillers in this condition contained at least one premise which was said to “NOT have” the property (see https://osf.io/fpx9k/ for all experimental materials and data, including premise typicality ratings).

### Procedure

Participants received instructions indicating that argument premises had been selected to be helpful for determining property extension (strong sampling) or generated randomly (weak sampling). In the strong sampling condition the text read:

*On each trial you will see three instances of living things that have a particular property. Note that the instances were deliberately chosen to best illustrate the variety of living things that have the property.*

In contrast, the weak sampling text emphasized the arbitrariness of the sampling process:

*On each trial you will see three instances of living things that have a particular property. We asked a student to open a book on plants and animals at random pages and note the first three living things they came across and whether or not those living things have the property in question. This means the information you receive may not be the most helpful for making your judgment—by chance, the student will sometimes select very dissimilar items, and sometimes very similar ones.*

They then saw 12 test trials (three diverse targets, three nondiverse targets, six fillers) in random order. On each trial, three premises were listed as having a shared novel property (or in fillers in the weak condition, some premises were shown not to have the property). Participants then rated the likelihood that all members of the conclusion category had the property (1 = *not very likely*, 7 = *very likely*; hereafter “argument strength”). For each participant, the property attached to each argument was drawn randomly from the 12 fictitious biological properties shown in Table 1e, with a different property used on each trial. After test, there was an attention check where participants had to identify the largest integer in a random sequence.

## Results

Ratings of argument strength were first averaged across the three diverse and three nondiverse targets for each participant in the strong and weak sampling groups. Mean group argument strength ratings and within-group standard errors for diverse and nondiverse arguments are plotted in Fig. 2c. There is a clear diversity effect: Properties shared by diverse premises were more likely to be generalized (*M* = 5.08, *SE* = .09) than properties shared by less diverse premises (*M* = 4.48, *SE* = .08, *BF*_10_ > 1,000, η_p_^2^ = 0.25).^1^ The sampling manipulation also influenced ratings of argument strength in the expected fashion, with participants in the weak sampling condition giving higher ratings overall (*M* = 5.23, *SE* = .11) than those in the strong condition (*M* = 4.33, *SE* = .11, *BF*_10_ > 1,000, η_p_^2^ = 0.15). Most importantly, there is strong evidence for an interaction: As predicted by our theoretical analysis, the diversity effect is attenuated under weak sampling relative to strong sampling (*BF*_10_ = 36.0, η_p_^2^ = 0.07). To confirm that the form of this interaction is indeed an attenuation of the diversity effect in the weak sampling condition (as opposed to a disappearance of the effect), we ran a Bayesian paired-samples *t* test for this condition alone and found strong evidence that the effect (*BF*_10_ = 136.0) still exists in this condition. Taken together, the higher overall level of generalization in the weak sampling condition and the fact that there is still a modest diversity effect in this condition suggest that people in this condition are not simply ignoring similarity among categories as a source of evidence, rather, they appear to assign different evidentiary value to this similarity.

**Fig. 3:**
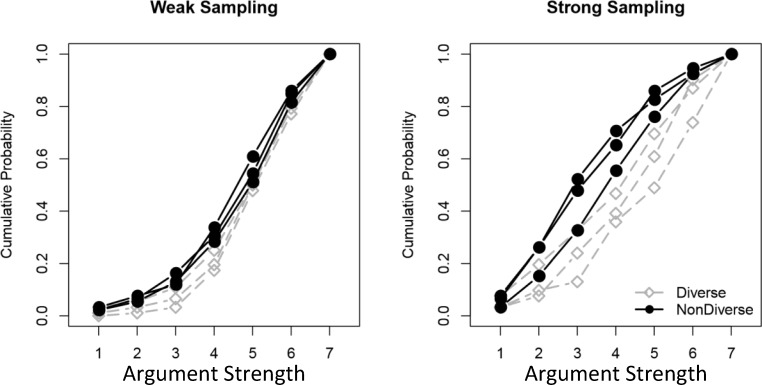
Cumulative distribution functions for argument strength ratings for all three diverse targets (black) and all three nondiverse targets (grey), plotted separately by condition. The *y*-axis plots the probability that the participant rated the argument as strong or less strongly than the value on the *x*-axis. In all cases, the grey lines are shifted to the right of the black lines, indicating that the diverse argument was rated as stronger. The tight clustering of all curves in the weak sampling condition (left) compared with the strong sampling condition (right) illustrates that the attenuated diversity effect is observed for all target arguments

**Fig. 4:**
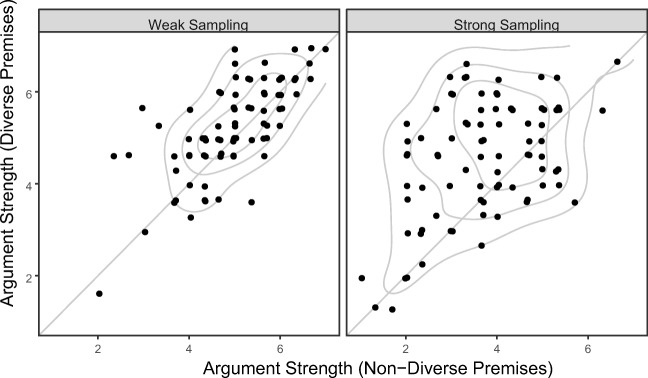
Scatterplots showing individual subject ratings. Each dot depicts a single participant, plotting the average rating they provided to the three nondiverse arguments (*x*-axis) against their average response to the three diverse targets (*y*-axis). Under weak sampling (left panel), the diversity effect is reflected by the fact that the distribution (contours) is shifted very slightly upwards from the diagonal line. Under strong sampling (right panel), a different pattern is seen: A majority of participants show a large diversity effect (points above the diagonal) whereas a minority show no diversity effect at all (dots lying on the diagonal)

Exploratory analysis suggested that the attenuation effect was consistent across the target arguments listed in Table 1, but heterogeneous across the 187 participants. Highlighting the homogeneity across arguments, Fig. 3 depicts the cumulative distribution functions over mean rated argument strength across participants in each sampling condition, plotted separately for each target argument. Where one argument received higher average ratings than another, its corresponding line appears to the right of the other. The fact that all of the grey lines (diverse arguments) appear to the right of all of the black lines (nondiverse arguments) illustrates the consistency of the diversity effect across arguments and between conditions (albeit attenuated under weak sampling). In contrast, Fig. 4 reveals individual differences across subjects in the strong sampling condition: the majority show large diversity effects (dots above the diagonal line) whereas a substantial minority (around 30%) show little to no diversity effect at all (dots near or below the diagonal line).

## Discussion

The effect of evidential diversity on property induction is one of the most widely replicated findings in the field of inductive reasoning. When introducing their Bayesian generalization model, Tenenbaum and Griffiths (2001) argued that it naturally accommodates the effect of diversity on inductive argument strength. In this article, we extend their analysis. We have shown empirically that the magnitude of the diversity effect depends on participants’ assumptions about how the evidence has been selected. As predicted by the Bayesian model, when led to believe strong sampling applies, a robust diversity effect appeared. However, when the context suggested that evidence was generated randomly (weak sampling), the diversity effect was attenuated.

Notably, this attenuation meant that overall ratings of property generalization were higher under weak than under strong sampling. As predicted, the largest effect of sampling was on inferences from evidence with low diversity where strong sampling prompted more restricted property generalization than weak sampling. In all crucial respects, the group empirical results were consistent with the ordinal predictions of the Bayesian model.

In regard to the generality of these effects, the predicted difference in the magnitude of the diversity effect under weak and strong sampling assumptions was obtained consistently across a variety of inductive arguments (see Fig. 3). Although our experiment only examined the results of a single operationalization of diversity (diverse vs. nondiverse premises), our simulation results (see Fig. 2a) shows that the same qualitative prediction about the effects of sampling assumptions holds across a range of possible levels of evidence diversity. The relationship between diversity effects and sampling assumptions should therefore be seen as a generic prediction of Bayesian inductive reasoning models. There was, however, suggestive evidence (see Fig. 4) for some heterogeneity in the effects of sampling assumptions across subjects. Although a majority in the strong sampling condition showed a robust diversity effect, some showed little effect of evidence diversity. This could reflect individual differences in belief in the cover story used to manipulate sampling assumptions, in knowledge of biological categories, or a more fundamental difference in the way that different individuals generate inductive hypotheses from diverse or nondiverse evidence (cf. Navarro et al., 2012; Ransom, Hendrickson, Perfors, & Navarro, 2018).

Our theoretical analysis and results make an important contribution by highlighting the central role played by sampling assumptions in important inductive phenomena like the diversity effect. Previous theoretical explanations of this effect (e.g., Heit, Hahn, & Feeney, 2005; Osherson et al., 1990) have focused on how diverse sample content promotes property generalization. The Osherson et al. (1990) model, for example, assumes that more diverse samples support broader generalization because they provide more coverage of the category of interest. In contrast, our approach suggests that the strength of the diversity effect depends on one’s assumptions about how premise information is selected—especially for the nondiverse samples. The fact that many previous studies (Feeney & Heit, 2011; Liew et al., 2018; Osherson et al., 1990) have demonstrated robust diversity effects in property induction without explicit manipulation of sampling assumptions suggests that strong sampling of the presented evidence may be the default for a majority of subjects. Notably, the assumption of strong sampling may be more widespread amongst adults than children. Rhodes, Gelman, and Brickman (2010) found that diverse evidence affected 5-year-olds’ inferences when it was presented by a knowledgeable domain “expert,” but not when it was presented by a domain “novice.” In contrast diverse evidence affected adults’ inferences in both conditions.

Our results add to a growing body of evidence highlighting the central role of sampling assumptions in determining what characteristics of an argument are deemed relevant to an inductive reasoning problem. For instance, when introducing the relevance theory perspective on inductive reasoning, Medin et al. (2003) demonstrated a premise nonmonotonicity effect, in which adding premises that share a distinctive relation (e.g., adding the premise black bears to grizzly bears) weakened belief that the premise properties generalized to a conclusion category (mammals). By casting this in an explicitly Bayesian framework, Ransom et al. (2016) showed that this effect arises naturally from a strong sampling assumption, and can be reversed when learners are encouraged to adopt a weak sampling perspective. A similar effect of sampling assumptions was found when learners were presented with combinations of positive and negative evidence (Voorspoels et al., 2015). Whether considering the quantity of evidence (Ransom et al., 2016), the kind of evidence (Voorspoels et al., 2015), or, as we show here, the diversity of evidence, the inferences people make are highly dependent on their beliefs about the sampling mechanisms involved.

This study highlights that category-based induction, like other tasks that involve drawing conclusions from data (Gweon, Tenenbaum, & Schulz, 2010; Shafto, Goodman, & Griffiths, 2014), is highly sensitive to sampling assumptions. It also raises questions about the precise sampling assumptions involved. Consistent with many previous studies (e.g., Gweon et al., 2010; Navarro et al., 2012; Ransom et al., 2016), we framed the question as one of “strong” and “weak” sampling. In many other studies, however, the key difference is characterized as a contrast between “helpful” (or pedagogical) and “random” sampling (e.g., Shafto et al., 2014; Voorspoels et al., 2015), suggesting that the social context is critical to these effects. Although there are some contexts where the distinction between strong or helpful sampling leads to different kinds of inferences (e.g., Navarro et al. 2012), the distinction is not crucial for understanding the diversity effect. More generally, the current work highlights a need to investigate how learners’ beliefs about evidence generation and transmission affect the range of other inductive phenomena (see Hayes & Heit, 2018, for a review) that have been central to building theories of category-based inference.

### Constraints on generality

Our work shows that the diversity effect in property induction depends, in part, on an assumption that the evidence presented in the experiment (i.e., the argument premises) was not selected randomly. Our target population for this work was adult reasoners. Because the diversity effect has been replicated in adult samples from a range of cultural backgrounds (e.g., United States, Belgium, Australia, China, Korea; see Choi, Nisbett, & Smith 1997; Medin et al., 2003), we expect that our results will have considerable cross-cultural generality. A constraint on generality is that we only examined diversity using categories and properties drawn from the domain of biology. It remains to be shown whether our results extend to reasoning about other domains (e.g., artifacts, social categories). Within the biological domain, we assume that our results apply to people with a modest amount of knowledge about biological kinds. However, they most likely do not apply to those with expert domain knowledge—who often do not show diversity effects when reasoning about objects within their area of expertise (e.g., Shafto & Coley, 2003).

## Electronic supplementary material

ESM 1 (DOCX 23.5 kb)

## Footnotes

1 Bayes factors were calculated using a mixed-effects Bayesian ANOVA, conducted using the BayesFactor package in R with default Cauchy priors.
