# Supplementary material for: The diversity effect in inductive reasoning depends on sampling assumptions
Source: Psychon Bull Rev. 2019 Jan 25;26(3):1043–50. doi: 10.3758/s13423-018-1562-2 (PMC6558053; doi:10.3758/s13423-018-1562-2)
Supplement: Supplementary file 1 — (DOCX 23.5 kb) [file 13423_2018_1562_MOESM1_ESM.docx]

**Supplementary Material: Details of Bayesian Model Simulation**

The key empirical prediction derived from our Bayesian framework is that the premise diversity effect in property induction will be larger under strong sampling assumptions than under weak sampling assumptions. It is important to show that this is a *generic* prediction of the framework. A simple simulation demonstrates this: we consider hypothesis spaces that consist of a set of categories with binary memberships, one of which is the superordinate to which all premise categories belong. We simulated 10,000 random hypothesis spaces for a domain consisting of 20 premises that can belong to 100 categories, assuming that on average each premise belongs to 5% of the possible categories (the qualitative predictions of the model do not depend on these specific values). That is, in each iteration we generated a new hypothesis space *H* described by a matrix with 20 rows (one per premise) and 100 columns (one per category). For each cell *H(i, j)* was either a 1, if premise *i* belonged to category *j*, or zero otherwise.

Within each simulated hypothesis space, we considered every possible combination constructed from arguments containing three premises. In each case, we calculated the “evidence for conclusion” (i.e., the posterior probability of the superordinate category) under both weak and strong sampling (without replacement).

We calculated premise diversity in the following way. First, we calculated the average pairwise similarity *s*(*x_i_*, *x_j_*) between pairs of premises, given by the number of categories to which both belong.^^[[1]](#footnote-1)^^ To illustrate, assume that for a given argument we have three premises (P1, P2, P3) corresponding to one unique combination of three premises drawn from the set of 20. For each pair of premises (Pa, Pb) we calculate the similarity by counting the number of columns in H where the entry for row Pa and row Pb both contain a 1. The result is a matrix as shown in the example below:

P1 P2 P3

P1 0 **3 1**

P2 0 0 **2**

P3 0 0 0

We calculate the similarity of the given argument by taking the mean of these numbers. In this example, the mean similarity of the premises = *M*(sim(P1,P2), sim(P1, P3), sim(P2, P3)) = (3 + 1 + 2) / 3 = 2. Given that we have 100 categories we now calculate the diversity of the premises in the argument as (100 – mean similarity) / 100 = (100 - 2) / 100 = 0.98.

Using the evidence for conclusion and premise diversity values calculated for each argument, we were able to construct curves relating these two quantities separately for strong and weak sampling (shown in Figure 2a of the main paper). To do so, it was necessary to aggregate the simulated data in two stages. Firstly, we collapsed all simulated arguments within a single hypothesis space by calculating the mean evidence for conclusion across all arguments with the same diversity value. We then repeated the process by collapsing arguments in a similar manner across all simulated hypothesis spaces. Aggregating the data via two stages in this way ensured that each simulated hypothesis space contributed evenly to the curves constructed. The result of this aggregation yielded the mean evidence for conclusion for a discrete number of diversity levels (typically 8-10 for the simulation parameters reported). We performed a LOESS (locally estimated scatterplot smoothing) regression (Cleveland & Devlin, 1988) to provide interpolated predictions when constructing the final curves.

To illustrate how our simulation can be used to generate predictions about empirical argument strength ratings when argument diversity and sampling assumptions are experimentally manipulated, we assumed that there was some latent “perceived” diversity (*d*) for the premises in the diverse conditions in our experiment, and a corresponding value (*n*) for premises in the non-diverse conditions. We estimated values for *d* and *n* separately by minimising the sum squared prediction errors of evidence for conclusion relative to mean rated argument strengths of the *diverse* and *non-diverse* conditions, respectively. For example, for a given candidate value *d'* of *d*, the sum squared prediction error is calculated across two predictions (one for *strong* *sampling* and one for *weak sampling*) taken from the simulation curves and the two corresponding empirical means (taken from the *diverse* × *strong* and *diverse* × *weak* conditions). The estimated values for *n* and *d* can be found in Figure 2a in the main paper, and predicted posterior probabilities based on these values are shown in Figure 2b. Given the differences between the response scales in the simulation and the experiment, and given that we cannot be sure about how closely our simulation matches the hypotheses considered by experimental participants, we make no claims about the veracity of the exact posterior values shown in Figure 2b. Nevertheless, the Figure illustrates how our simulation can be used to derive *qualitative* predictions about ratings of diverse and non-diverse arguments in the respective sampling conditions.

**Reference**

Cleveland, W. S., & Devlin, S. (1988). Locally weighted regression analysis by local fitting. *Journal of the American Statistical Association, 83*, 596– 640.

1. Code, data and materials available at <https://osf.io/fpx9k/> [↑](#footnote-ref-1)
